# Comparative Study on Reagents Involved in Grape Bud Break and Their Effects on Different Metabolites and Related Gene Expression during Winter

**DOI:** 10.3389/fpls.2017.01340

**Published:** 2017-08-04

**Authors:** Muhammad Khalil-Ur-Rehman, Wu Wang, Yan-Shuai Xu, Muhammad S. Haider, Chun-Xia Li, Jian-Min Tao

**Affiliations:** College of Horticulture, Nanjing Agricultural University Nanjing, China

**Keywords:** quiescence, metabolites, gene expression, hormone, antioxidants

## Abstract

To elucidate promoting and inhibiting effects of hydrogen cynamide (HC) and abscisic acid (ABA) on quiescence release of grape buds, physiological and molecular approaches were used to explore the mechanisms of quiescence based on metabolic and gene expression analysis. Physiological and molecular mechanisms involved in bud quiescence of grape were studied before and after application of HC, ABA, and ABA-HC. The data showed that ABA inhibited proclamation of quiescence in grape buds and attenuated the influence of HC. Bud quiescence was promoted and regulated by HC and ABA pre-treatment on buds of grape cultivar “Shine Muscat” with 5% HC, 100 μM ABA and combination of ABA-HC (5% HC+100 μM ABA) during quiescence under forcing condition. Exogenous application of ABA elevated superoxide dismutase (SOD), peroxidase (POD) and ascorbate peroxidase (APX) related specific activities, while catalase (CAT) activity was increased during initial period of forcing and then decreased. The concentration of plant growth hormones including gibberellins (GA) and indole acetic acid increased by HC application but decreased the ABA contents under forcing condition. ABA increased the fructose content during quiescence under forcing condition while sucrose and total soluble sugars peaked in HC treated buds as compared to control. Genes related to ABA pathway, protein phosphatase 2C (PP2C family) were down regulated in the buds treated with HC, ABA and ABA-HC as compared to control while two genes related to GA pathway (GID1 family), out of which one gene showed down regulation during initial period of forcing while other gene was up regulated in response to HC and ABA-HC treatments as compared to control. Exogenous ABA application up regulated genes related to antioxidant enzymes as compared to control. The gene probable fructose-bisphosphate aldolase 1, chloroplastic-like, was up regulated in response to ABA treatment as compared to control. Analysis of metabolites and related gene expression pattern would provide a comprehensive view of quiescence after HC, ABA, and ABA-HC treatments in grape buds which may helpful for ultimate improvement in table grape production.

## Introduction

Grape (*Vitis vinifera L*.) is an important fruit crop worldwide and table grapes are considered as the major horticultural crop of China due to their Chinese origin (Wang et al., 1998; He, 1999; Du et al., 2008). Several value added products are prepared from grape fruit and investments are being made to develop new viticulture regions (Hoff et al., 2017; Imran et al., 2017). The area under grape plantation is increasing promptly in China, standing fourth in the World according to the OIV statistical report on world Viti-viniculture (OIV, 2012).

In woody perennials, bud dormancy is an intricate process that endures plants in response to abiotic stress (extremes events drought, cold, and heat) which are characterized by inhibited growth, apprehended cell division as well as obstructed respiratory and metabolic activities (Arora et al., 2003). Seasonal environmental changes adopted by woody perennials during dormancy promote their survival under unfavorable environment and ensures simultaneous blooming in the orchard which ultimately improves fruit production under such conditions.

A period of low temperature is often termed as “winter chilling” is required in temperate perennial species for proclamation of the buds from endodormancy (ED). In numerous grape and apple cultivars, diminishing photoperiod along with warm winters can persuade the bud into ED (Bound and Jones, 2004; Kühn et al., 2009). Proclamation of dormancy poses a key hindrance to commercial viticulture in regions with warm winter. Insufficient cold accumulation during this period generally results in non-uniform flowering and reduced fruit set. The grape buds in southeast China can achieve their chilling requirement at the end of February and bloom in the following spring (Khalil-Ur-Rehman et al., 2017). Therefore, to allow coordinated and early production of economically viable yields, artificial substitutes are required for chilling to avoid prolonged dormancy in these regions (Zheng et al., 2015). In southern China (southeast and southwest China), grape cultivation is being practiced under rain shelter green house and the dormancy breaking reagents are generally applied to mitigate the adversarial effects of insufficient chilling to vines. The application of hydrogen cynamide (HC) is an effective practical mean for dormancy release, widely used in the global table grape industry (Lavee and May, 1997; Or, 2009). There are evidences that dormancy break and oxidative processes in grape buds are interconnected (Or et al., 2002; Pérez and Lira, 2005). HC has the ability to suppress expressional activity of the CAT, leading to a transient peak of hydrogen peroxide (H_2_O_2_), which regulates the release of ED and bud break (Pérez and Lira, 2005; Halaly et al., 2008). Moreover, application of HC stimulated the temporary elevation in H_2_O_2_ levels and rapidly up regulates certain genes associated with oxidative stress (Pérez et al., 2008; Ophir et al., 2009). HC application up-regulated the sucrose synthase expression, as well as that of pyruvate decarboxylase, a sucrose non-fermenting (SNF)-like protein kinase, GDBRPK and alcohol dehydrogenase (Halaly et al., 2008; Ophir et al., 2009). As stated above, it indicates that the HC application initiates the regulation of oxidative stress which might have its key role in bud break. Light plays an important role in biosynthesis of hormones like GA’s and may decrease the accessible key metabolites for plant growth and development (Li et al., 2017).

Apart from external factors like light, temperature, nutrition, and water supply; internal factors such as carbohydrate levels, hormones and enzyme activity are also involved in bud break and dormancy release. A crosstalk between grape bud dormancy and plant hormones along with the bud break is a critical dilemma for grape production. Previously, Guevara et al. (2008), Mornya and Cheng (2011), Okay et al. (2011) examined the endogenous hormonal change during bud dormancy release process and demonstrated that regulation and control, occurrence and termination of dormancy were regulated by hormones. Different levels of endogenous abscisic acid (ABA) are linked with development and release of apical buds dormancy in plants (Powell, 1987).

The ABA is regarded as the key hormone during ED that uplifts the bud dormancy regulation (Cooke et al., 2012). The ABA level tend to increase during fall season, which results in shoot growth cessation, apical buds set promotion and bud dormancy induction. Similar study on poplar has revealed that ABA levels were peaked during the ceased growth of apical buds complemented with ABA synthesis-related genes (ABA1, NCED3, and ABA2) and components of ABA signal transduction (PP2C, ABAI1, and AREB3) (Arora et al., 2003; Ruttink et al., 2007). The elevated ABA level, accompanied by higher expression of VvNCED1 during grape bud dormancy induction and maintenance; yet, ABA catabolites increased and endogenous ABA levels declined when buds attained adequate chilling accumulation (Zheng et al., 2014). Likewise, gibberellins (GAs) are also pivotal to dormancy induction and release after chilling contentment (Schrader et al., 2004). The exogenous application of GAs can also stimulate the dormancy break in many angiosperms (Looney, 1997).

Carbohydrate reserves in plants undertake seasonal variations; they collect late in perennial structure during the growth period and are utilized later during bud growth recommencement (Zapata et al., 2004). Therefore, it seems likely that carbohydrate reserves are the key energy source that arises during dormant period for the metabolic changes and spring bud break. Report suggested that in plants total soluble sugars (TSSs) increased at the beginning of cold conditions, peaked at full cold hardiness and decline during deacclimation (Sakai and Larcher, 2012). Many researchers suggested that variation in enzyme activity as a signal at dormancy ending and beginning of new growth (Marquat et al., 1999; Citadin et al., 2002). These contrasting reports sometimes underscore the need for further research into the roles of hormones, sugars, antioxidant enzymes and related gene expression level during grape bud dormancy. Previous studies focused the variations in molecular and physiological mechanisms involved in grape bud dormancy separately (Ben Mohamed et al., 2012; El-Yazal et al., 2014; Zheng et al., 2015), no attempt has yet been made to simultaneously investigate the molecular and physiological mechanisms of grape bud dormancy.

Therefore, this study was carried out to understand how changes occurred in the levels of hormones, sugars, antioxidant enzymes and related gene expression after application of HC, ABA, and ABA-HC and effect the timing and degree of dormancy and the related processes. Additionally, this study was endeavored to explore the influence of HC, ABA-HC, and ABA and their possible mechanism at physiological and molecular level under forcing condition. The present study will help to get better understanding about underlying physiological and molecular mechanisms which may helpful for ultimate improvement in table grape production.

## Materials and Methods

### Plant Materials

A Japanese Cultivar ‘Shine Muscat’ (*Vitis labruscana Bailey × V. vinifera L*.) used as material was collected from 6 years old plants supplemented with drip irrigation in sandy soil under rain shelter protected with polyvinyl film, at vineyard of Nanjing Agricultural University located in Tangshan Valley, Nanjing, China. Vines were not pruned or conventionally treated with chemicals during sample collection period. Canes were collected on January 18th, 2016. Each separated cane carrying 10 buds (positions 3–12) were moved to laboratory for treatments. Each cane was cut into single node cuttings, randomly mixed and a group of 10 cuttings were prepared for each treatment, respectively. Three replicates (thirty cuttings in each replicate) were used for each treatment under forcing condition.

### ABA Application

Cuttings were treated and immersed in glass bottles with 100 μM ABA (Sigma–Aldrich, Co., United States) by adding 0.02% (v/v) Triton X-100 (Sigma–Aldrich, St. Louis, MO, United States). The bottles were moved to growth chamber and forced at 25 ± 1°C under 14 h/10 h light dark condition. Cuttings were shifted to tap water after 48 h of incubation in ABA. Similarly, the cuttings for control treatment were treated with 0.02% solution of Triton X-100.

### Induction of Dormancy Release by HC and ABA-HC

After 48 h of pre-treatment with water or ABA (100 μM), subsequent treatment was applied considering as 0 h for sampling and bud break monitoring. The treated groups of cuttings were returned to glass bottles having water and nurtured under forcing conditions as mentioned above for further 28 days for monitoring of bud break. The cuttings in control group were sprayed with tap water, while, cuttings were treated with 5% (v/v) ‘Dormex’ (SKW, Trostberg, Germany) in HC treatment (Zheng et al., 2015). In ABA treatment, 100 μM of ABA was used to treat the cuttings. The 0.02% Triton X-100 was used as wetting agent in preparation of all solutions. Cuttings were first treated for 48 h with 100 μM ABA and then with 5% HC for combined ABA-HC.

Bud break was monitored 10, 14, 18, 22, 26, and 30 days after treatment under the above mentioned forcing conditions. For antioxidants, sugars, hormone and gene expression analyses, buds were sampled at 12, 24, 48, 96 h, 7 and 14 days. The sampled buds were first frozen in liquid nitrogen and then kept at -80°C.

### Extraction and Quantification of Hormones

The determination of endogenous indole acetic acid (IAA), GA3 and ABA were carried out using liquid chromatography–tandem mass spectrometry (LC–MS/MS) method as described by Müller and Munné-Bosch (2011). Peaks were automatically detected based on the retention time and MRM transition. Peak areas were normalized relative to the internal standards to account for variations in sample preparation and analysis. Contents of hormones were calculated according to the calibration curves created with the authentic samples using the software Analyst and Multi Quant (AB SCIEX, Framingham, MA, United States).

### Determination of Carbohydrate Content

Sucrose and fructose contents in buds were assayed according to the methodology used by Hendrix (1993). The soluble sugar content was measured using spectrophotometer and absorbance was measured at 620 nm using an anthrone reagent as proposed by Bates et al. (1973).

### Enzyme Activities Assay

The activities of catalase (CAT) and peroxidase (POD), ascorbate peroxidase (APX), and SOD were assayed according to the methodologies proposed by Giannopolitis and Ries (1977), Nakano and Asada (1981), and Wang et al. (1991) respectively. The activities of CAT, POD, and APX were determined from the measurement of absorbance at 240, 420, and 290 nm, respectively.

### Hydrogen Peroxide (H_2_O_2_) Content Determination

The H_2_O_2_ content (nmol g^-1^ fresh weight) from grapevine buds was determined as described by Loreto and Velikova (2001). The absorbance was measured using spectrophotometer at 390 nm.

### Quantitative Real Time PCR

Total RNA from grape buds was isolated using Trizol reagent (Invitrogen, United States) as instructed by manufacturer. For each treatment three biological replicates were prepared. A Nano-drop spectrophotometer (ND-1000, NanoDrop Technologies, Wilmington, DE, United States) was used for quantification of RNA. Quality of RNA was checked using a 2100 Bio-analyzer (Agilent Technologies, Santa Clara, CA, United States) according to protocol suggested by the manufacturer. For first-strand cDNA synthesis 1 μg RNA was reverse transcribed using the SYBR PrimeScript RT-PCR Kit II (TaKaRa, Japan) with 2.5 μM oligonucleotide dT primer and 5 μM random hexamer priming method according to the conditions recommended by the manufacturer. Prior to quantitative real time quantitative PCR (qRT-PCR) reverse transcribed products were diluted 10 times in water. For qRT-PCR aliquots of cDNA were used as template. Reactions were set up with Power SYBR^®^ Green PCR Master Mix (Applied Biosystems) according to the manufacturer’s instructions in a total volume of 20 μl and 0.3 μM of each primer. The ABI Step One Plus Real Time PCR System (Applied Biosystems) was used to detect amplification levels and was programmed for an initial step at 95°C for 2 min, followed by 40 cycles of 15 s at 95°C and 1 min at 60°C. All reactions were run in duplicate or triplicate and average values were calculated. Quantification was performed with at least two independent experiments. The housekeeping Actin gene (VvActin) was used as an endogenous control. Relative expression levels of target genes and SD values were calculated using the 2^-ΔΔCt^ method (Livak and Schmittgen, 2001). The sequences of forward and reverse primers for each gene are listed in Supplementary Table S1.

### Statistical Analysis

The data of HC, ABA, and ABA-HC treatments were subjected to analyses of variance (ANOVA) to evaluate the variation during quiescence period using GLM procedure in IBM SPSS Version 19.0 (IBM Corporation, Armonk, NY, United States). Tukey’s HSD *post hoc* test (*p* = 0.05) was used to compare the means. Sigma Plot 10.0 (Systat Software, Inc., Chicago, IL, United States) was used for plotting graphs.

## Results

### Influence of ABA-HC, HC and ABA on Bud Dormancy Release

**FIGURE 1:**
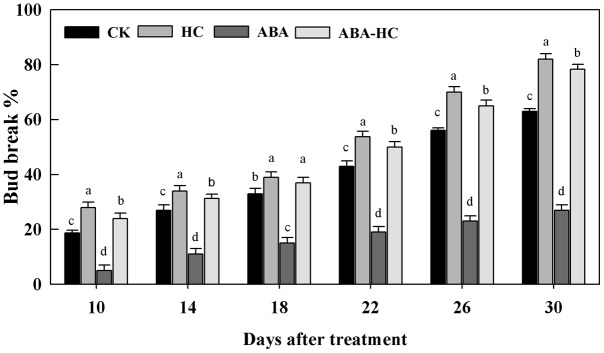
Effect of HC, ABA, and ABA-HC application on bud break of single node cutting of cv. ‘Shine Muscat’ grape forced for 30 days under 25 ± 1°C 14/10 h dark/light conditions. Control buds were treated with water. Verticle lines above the means bars indicate standard error (*n* = 3; *p* = 0.05) using Tukey’s HSD *post hoc* test. a–d represents significant difference between treatments.

The quiescent buds responses to application of ABA, a famous inducer of quiescence release (HC) and collective treatment with HC, ABA and (ABA-HC) or water were compared. Supplementary Figure S1 shows the timing and treatment combination of the experiment. HC treatment led to the estimated development of bud quiescence release compared to the control. However, ABA had a considerable repressive effect on quiescence release of grape buds (**Figure 1**). Using100 μM ABA for 48 h incubation of single node cuttings which resulted in reduction of 16, 18, 24, and 15% in bud-break percentage compared to the control at 10, 14, 18, and 22 days after treatment, respectively. The treatments applied on the buds may be confounded with hypoxic condition.

### Influence of ABA, ABA-HC and HC Treatment on Endogenous Hormone Contents of Grape Buds

**FIGURE 2:**
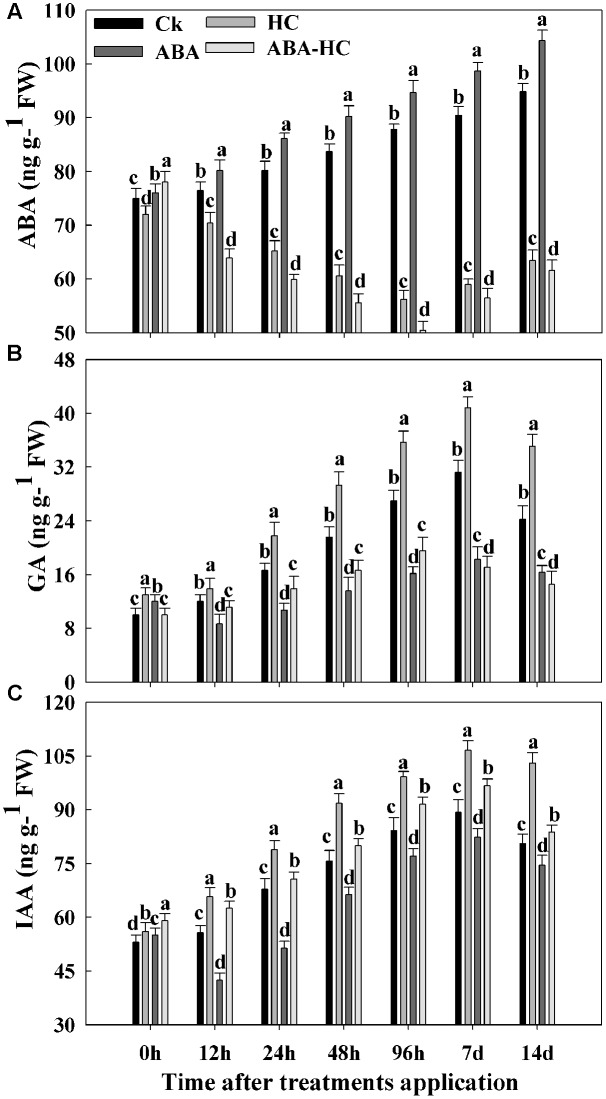
Effect of HC, ABA, and ABA-HC on contents of **(A)** ABA, **(B)** GA, and **(C)** IAA in the buds of cv. ‘Shine Muscat.’ The mesurements in treated and control bud samples were made at 0, 12, 24, 48, 96 h, 7 and 14 days after treatement. Verticle lines above the means bars indicate standard error (*n* = 3; *p* = 0.05) using Tukey’s HSD *post hoc* test. a–d represents significant difference between treatments.

The endogenous GA, ABA, and IAA levels were determined in, ABA-HC, HC, and ABA treated and control buds. The sampling was carried out at 12, 24, 48, 96 h, 7 and 14 days after treatment (**Figure 2**). Compared with control ABA contents decreased initially after HC treatment and then slight increase was observed in grape buds under forcing condition. ABA treatment increased endogenous ABA contents in grape buds under all treatments and time points while decreasing trend of ABA contents were observed in HC and ABA-HC treatment compared with control. Maximum GA (40.85 ng^-1^ g FW) and ABA contents (104.29 ng^-1^ g FW) were recorded in samples treated with HC and ABA at 7 and 14 days while maximum IAA contents (106.53 ng^-1^ g FW) were recorded at 7 days samples treated with HC as compared to control.

### Enzymatic Antioxidant Activities

**FIGURE 3:**
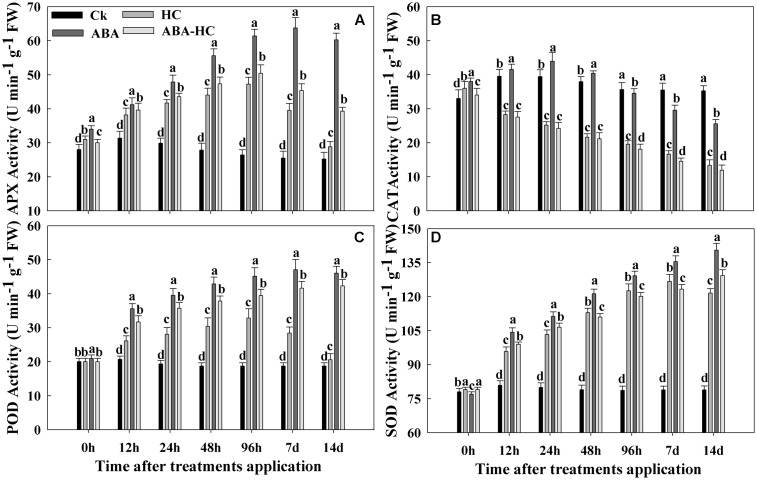
Changes in the activities of antioxidant enzymes **(A)** APX, **(B)** CAT, **(C)** POD, and **(D)** SOD in the buds of cv. ‘Shine Muscat’ treated with HC, ABA and ABA-HC or water and sampled at 0, 12, 24, 48, 96 h, 7 and 14 days after treatement. Verticle lines above the means bars indicate standard error (*n* = 3; *p* = 0.05) using Tukey’s HSD *post hoc* test. a–d represents significant difference between treatments.

After exogenous application of HC, ABA and ABA-HC on the buds, CAT activity was repressed by ABA-HC and HC application and it was lower in both treatments than in control during sampling period (**Figure 3**). CAT activity was high before HC treatment but after HC application this activity fell down quickly from 12 h to 14 days. As a result of ABA treatment, CAT activity increased initially and then decreased. In control buds CAT activity stayed stable after initial slight increase.

Unlike CAT activity, an increase in APX activity under ABA, HC, and ABA-HC treatment was observed (**Figure 3**). In fact, in ABA, HC, and ABA-HC treated buds APX activity increased rapidly. On the contrary in untreated buds APX activity declined and attained its lowest level after 7 days of treatment and remained stable afterward. POD activity increased in buds treated with ABA during all time points while the buds treated with ABA-HC and HC showed increasing trend. In control buds, the POD activity reduced initially and remained stable for rest of the examination period.

The time course of SOD activity in treated buds showed transient increase during the experimental period in three treatments (**Figure 3D**). Compared to that of control, SOD activity first increased and then decreased in HC treated buds. Whereas, ABA treatment elevated SOD activity during the experimental period compared to control.

### Carbohydrate Contents

Carbohydrate contents in grape buds treated with and without HC, ABA, and ABA-HC were investigated under forcing condition. HC treatment resulted in an increase of sucrose content as compared to control. Maximum sucrose contents (42.66 mg^-1^ g DW) were recorded after 96 h in buds treated with HC followed by ABA-HC treatment (36.40 mg^-1^ g DW). An increase in sucrose contents were observed in untreated buds after 96 h treatment.

**FIGURE 4:**
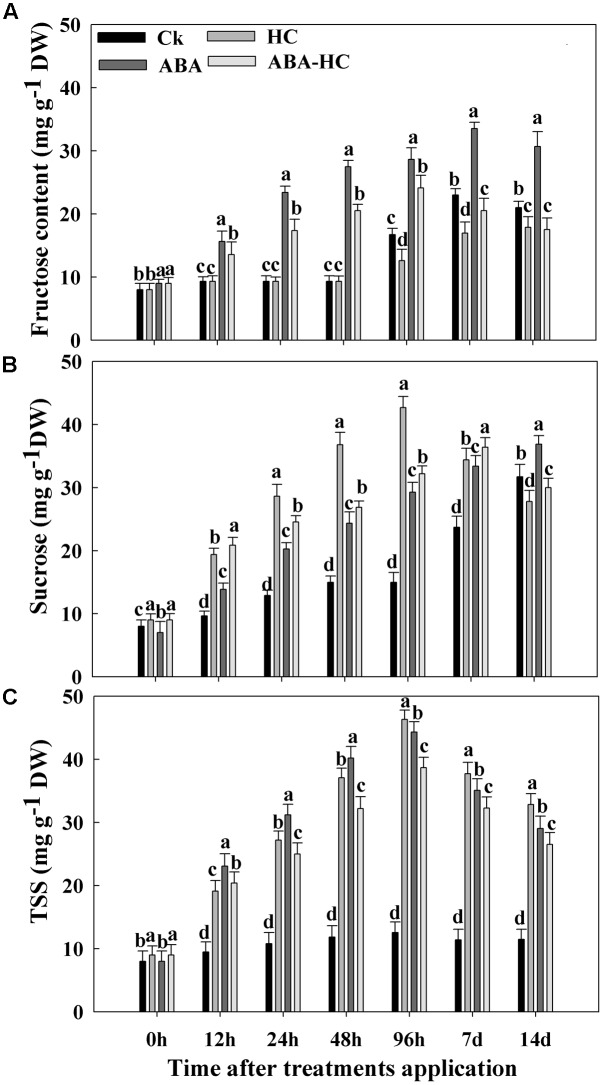
Changes in carbohydrate **(A)** Fructose, **(B)** Sucrose, and **(C)** TSS concentration in the buds of cv. ‘Shine Muscat’ treated with HC, ABA and AB-HC or control and sampled at 0, 12, 24, 48, 96 h, 7 and 14 days after treatement. Verticle lines above the means bars indicate standard error (*n* = 3; *p* = 0.05) using Tukey’s HSD *post hoc* test. a–d represents significant difference between treatments.

Abscisic acid treatment increased the fructose content than other treatments. Higher fructose contents (33.50 mg^-1^ g DW) were recorded at 7 days compared to control (**Figure 4**). HC increased the TSSs contents but no noteworthy variation was observed between HC and ABA treatments compared to control. Higher TSS contents (42.66 mg^-1^ g DW) were observed in buds treated with HC at 96 h compared to control (**Figure 4**).

### Influence of ABA-HC, HC and ABA on H_2_O_2_ Content of Grape Buds

**FIGURE 5:**
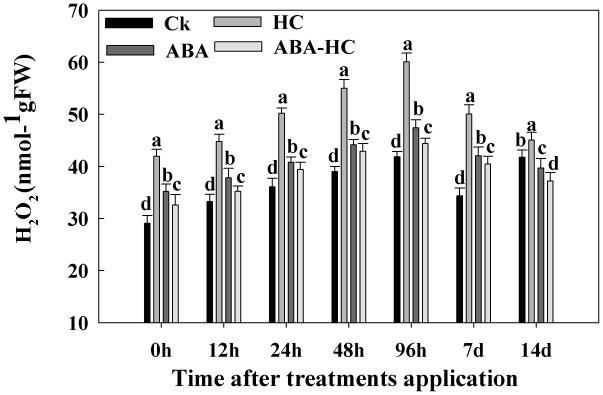
Effect of HC, ABA, and ABA-HC on H_2_O_2_ content in the buds of cv. ‘Shine Muscat’ treated with HC, ABA and AB-HC or control and sampled at 0, 12, 24, 48, 96 h, 7 and 14 days after treatement. Verticle lines above the means bars indicate standard error (*n* = 3; *p* = 0.05) using Tukey’s HSD *post hoc* test. a–d represents significant difference between treatments.

The effects of HC, ABA, and ABA-HC on H_2_O_2_ content in dormant buds of grape are shown in **Figure 5**. The buds treated with HC showed increasing trend of H_2_O_2_ as compared to control. Maximum content of H_2_O_2_ (60.06 nmol g^-1^ FW) was observed in the buds treated with HC after 96 h treatment followed by ABA treatment (47.33 nmol g^-1^ FW). An increase was observed in H_2_O_2_ content of untreated buds after 7 days treatment.

### Influence of ABA-H, HC and ABA on Gene Expression Related to Hormones, Antioxidants, and Carbohydrates

**FIGURE 6:**
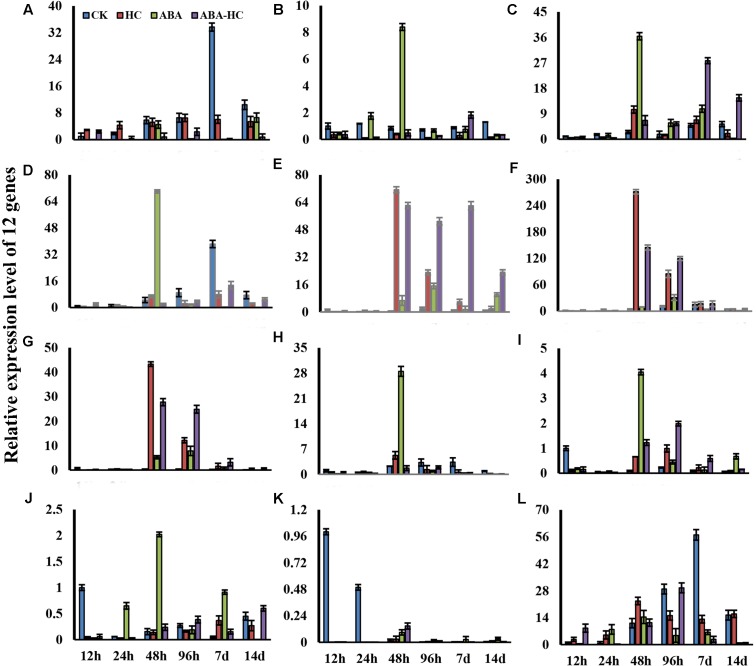
Relative Expression level of 12 genes **(A)**. Gene ID: LOC100245705, Gene name: probable glutathione peroxidase **(B)**. Gene ID: LOC100262354, Gene name: probable fructose-bisphosphate aldolase 1, chloroplastic-like **(C)**. Gene ID: LOC100249258, Gene name: peroxidase 46 **(D)**. Gene ID: LOC100259748, Gene name: 2-Cys peroxiredoxin 2-Cys peroxiredoxin **(E)** Gene ID: LOC100255710, Gene name; probable carboxylesterase 8 **(F)** Gene ID: LOC100260853, Gene name: probable carboxylesterase 8 **(G)** Gene ID: LOC100244514, Gene name: transcription factor bHLH35 **(H)** Gene ID: LOC100264830: Gene name: transcription factor UNE12 **(I)** Gene ID: LOC100233013, Gene name: L-ascorbate peroxidase 2, cytosolic **(J)** Gene ID: LOC100262779, Gene name: protein TIFY 8 **(K)** Gene ID: LOC100254174, Gene name; probable protein phosphatase 2C 14 **(L)** Gene ID: LOC100253351, Gene name, protein kinase and PP2C-like domain-containing protein. The values represents the mean ± SE of three biological and two technical repeats. Relative expression level were presented for HC. ABA, ABA-HC and control buds sampled at12, 24, 48, 96 h, 7 and 14 days after treatment. a–d represents significant difference between treatments.

To analyze the molecular mechanism underlying HC, ABA, and ABA-HC induced bud quiescence of grape; qRT-PCR analysis was carried out to observe the expression of genes related to hormones, antioxidants and carbohydrates (**Figure 6**). In our study genes related to ABA (PP2C family) were down regulated in all treatments except (Probable protein phosphatase 2C 14; Protein kinase and PP2C-like domain containing protein) peaked in 48 h after in response to HC and other treatments as compared to control (**Figure 6L**). The expression pattern of two genes related to GA (GID1 family) was observed. Gene (probable carboxylesterase 8) showed down regulation at 12 and 24 h treatments while peaked at 48, 96 h and 7 days after treatments in response to HC and ABA-HC treatments as compared to control while (carboxylesterase 8-like) gene showed up regulation at 48 and 96 h treatments in response to HC and ABA-HC as compared to control. Genes related to auxin family (transcription factor bHLH35, transcription factor UNE12, and protein TIFY 8) showed up regulation at 48 h in response to HC and ABA treatments as compared to control while, showed down regulation at 12, 24 h and 14 days in response to other treatments. Exogenous ABA up regulated three genes related to antioxidants enzymes, peroxidase 46, 2-Cys peroxiredoxin and L-ascorbate peroxidase 2, cytosolic at 48 h as compared to control while, no significant difference was observed in probable glutathione peroxidase 2 at 7 days in control treatments. The expression level of genes related to sugar metabolism were observed during bud dormancy of grape. Gene (probable fructose-bisphosphate aldolase 1, chloroplastic-like) showed up regulation in response to ABA treatment as compared to control.

## Discussion

Grape is considered as most popular and common fruit crop globally. Dormancy/quiescence in perennial plants is highly programmed and a very complex mechanism to survive with and thrive under harsh ecological conditions. A recent review reported the dormancy and quiescence as synonymically same but physiologically different terms (Considine and Considine, 2016). For dormancy breaking various chemical compounds are generally applied to start ED release of fruit buds of deciduous plants in subtropical or warm winter regions. In this study, artificial quiescence release induction of grape buds by HC, ABA, and ABA-HC and bio-chemical as well as metabolic changes involved using single node cuttings were observed under controlled environment. Our results depicted that the application of 5% HC solution advanced and improved bud break (**Figure 1**). Higher percentage of grape bud break treated with HC was observed after 1 month of forcing than that of other treatments and control, proposing a homogenized chemically initiation of dormancy break which does not happen in natural conditions (Ben Mohamed et al., 2010). During dormancy, a lot of changes in buds in some chemical components mainly in the endogenous hormones contents such as ABA, GA and IAA, found to take place for playing an important role in regulating dormancy and bud break.

To respond environmental signals plants are facilitated by endogenous hormones (Horvath et al., 2003). In many developmental processes endogenous GA’s plays a role and have been confirmed to take part in regulation of dormancy (Wang et al., 2006). It is identified in dormancy of tress like apple; control of bud dormancy regulation is interceded by changes in hormone signaling (Horvath et al., 2003; Rohde et al., 2007).

Our results showed that higher GA contents were observed in buds treated with HC compared with other treatments during quiescence and are in concurrence with studies stated by Duan et al. (2004) and El-Yazal et al. (2014) in **Figure 2**. During the experimental period, the ABA contents of HC treated buds decreased while higher level was recorded in buds treated with ABA (**Figure 2A**). Zheng et al. (2015) reported that by promoting ABA degradation HC treatment escorted to reduce endogenous ABA level, slowing down ABA synthesis or both. Higher contents of endogenous GAs and IAA which recorded in grape buds treated with HC are consensus with the previous study reported by Kuroi (1985). Study revealed that the single hormone whose endogenic concentration retorted to minor concentration of HC was IAA (Guevara et al., 2008). In control plants, measured IAA levels were observed to be declined, while in HC treated plants indicated to improve. This might show that H_2_O_2_ with IAA perhaps collectively was along with the preliminary responses of the plant toward HC application and elevated IAA level might activate other hormonal changes. Fuchigami and Nee (1987) proposed that by diminishing the endogenous ABA in buds, HC breaks dormancy and also its association in the combination of the thiol group, which is thought to be implicated in dormancy release. Prior to quiescence release subsequent to HC treatment was escorted by variation in different antioxidants like CAT, POD, and APX (**Figure 3**). Catalase activity, identified to have higher resemblance for H_2_O_2_ was inhibited during forcing. The simultaneous rise in H_2_O_2_ level due to inhibition of both the enzyme activities and genes expressions was stated in grape buds subsequent to the HC treatment (Pérez and Lira, 2005; Halaly et al., 2008). In this study the initiation of APX and POD activities following HC treatment were observed may be moderately elucidated by the elevated H_2_O_2_ levels of in bud cells ensuing due to low activity of catalase. This initiation was not stable and viewed in untreated buds. Moreover, these changes in enzyme activities were rapid and short lived and positively associated with higher and earlier bud break percentages that HC application exerts. Recent studies have shown that ABA protects plants from various types of abiotic stresses by enhancing antioxidant ability (Ding et al., 2010; Wang et al., 2011). In current study, we examined the possible regulatory role of exogenous ABA on APX, CAT, SOD, and POD activities. The working hypothesis was that via anti oxidative properties, ABA can alleviate oxidative stress in plants. Under drought stress, ABA pre-treatment induced SOD enzyme activity (Wang et al., 2011). In our study, ABA treatment elevated SOD activity during the experimental period compared with control. In plants, a number of enzymes are related to H_2_O_2_ metabolism mainly CAT, APX, and POD. ABA pre-treatment activated SOD and POD under abiotic stress (Wang et al., 2011). In our study ABA elevated the SOD, POD, and APX activities while reduced the CAT activity under forcing condition. Taken together, these results suggested that pre-treatment with exogenous ABA increased the capability of grape buds to scavenge excessive H_2_O_2_ under forcing condition mainly via SOD and POD activities. In this study, we explored the influence of HC, ABA, and ABA-HC on carbohydrate metabolism in endodormant buds. The increase in bud break percentage in response to HC application was escorted with significant variations in carbohydrate metabolism in the buds.

During growth resumption, buds which acts as a strong sink, uses stored carbohydrates (Ben Mohamed et al., 2010). Soluble sugars’ accumulation correlates well with bud ED release (Marquat et al., 1999). In many cases, soluble sugars do not function as simple nutrients to sustain growth but also as indicator that affect and control bud growth and development (Roitsch and González, 2004; Chao and Serpe, 2010). Hence, the transient soluble sugars accumulation, recorded here, acts a possible key role by reducing the osmotic potential which triggers the events leading to bud ED release, as suggested in dormant onion bulbs (Benkeblia et al., 2005). When bud break on treated cuttings started after about 10 days of forcing, we noticed a rapid consumption of soluble sugars in both treated organs indicating that metabolic activity of the buds has been intensified. In contrast, the sugars accumulation and consumption were less rapid and less evident in untreated buds reflecting the differences in bud development which was more advanced and more intense in HC treated buds but delayed and uneven in controls. It is, therefore, possible that the differences in the timing and extent of soluble sugars accumulation and consumption processes between HC-treated and control cuttings are responsible for the observed consequences of HC application on bud break timing and percentage. ABA has been involved in dormancy regulation and plays significant role in several plant reactions to abiotic stresses (Bray, 2002). In this study we evaluated contents of carbohydrate in ABA treated grape buds under forcing condition. ABA treatment resulted in an increase of fructose, sucrose, and TSS contents. The increase in soluble carbohydrates was observed after 100 μM ABA treatment as compared to control (**Figure 4**) indicating the effect of application of exogenic ABA on sugar contents of plant are mostly dependent on the concentration of ABA applied (Meng et al., 2008). Furthermore, all measured soluble carbohydrates increased in the buds treated with ABA. Further studies should be conducted to get more insights about the consequences of exogenic ABA application on carbohydrate metabolism during bud quiescence. ABA signaling in *Vitis vinifera* have been characterized and identified recently. In the current study, analysis presented the influence of ABA, HC, and ABA-HC on the level of gene expression. In previously reported studies (Yoshida et al., 2015) the expression level of two transcripts Vv PP2C4 and Vv PP2C were observed. These transcripts were significantly reduced in response to HC treatment. Our results showed that genes related to ABA (PP2C family) (Protein kinase and PP2C-like domain containing protein; Probable protein phosphatase 2C 14) were down regulated in all treatments except peaked in 48 h after in response to HC and other treatments compared to control (**Figure 5**). These variations might reveal a reaction to a low ABA level and provided as an further confirmation for ABA related changes in response to HC. Report revealed that calcium signaling is implicated in the mechanism of bud dormancy release in grape (Pang et al., 2007). In our study, the expression pattern of two genes related to GA (GID1 family) were observed. Different gene expression pattern after different treatments might play an imperative role in grape bud dormancy regulation. Previous reports revealed that Mn-SOD strongly responds to oxidative stress and ABA induces its expression (Bueno et al., 1998; Li et al., 2009). In current study, exogenous ABA notably up-regulated three genes related to antioxidant enzymes; peroxidase 46, 2-Cys peroxiredoxin and L-ascorbate peroxidase 2, cytosolic at 48 h as compared to control, while no significant difference was observed in probable glutathione peroxidase 2 at 7 days in control treatments. ABA induced expression of antioxidants genes may contribute in dormancy maintenance and regulation of grape buds. Sugars play a potential signaling role in dormancy status. In crown buds of leafy spurge, the endodormant period was noticed by particular cell expansion and xyloglucan endotransglycosylase (XET) up-regulation. Higher XET expression in poplar’s cambial The expression level of genes related to sugar metabolism was observed during bud dormancy of grape in present study. Gene (probable fructose-bisphosphate aldolase 1, chloroplastic-like) showed up-regulation in response to ABA treatment as compared to control. The results suggest that genes related to carbohydrate metabolism might have a significant role in grape quiescence regulation.

## Conclusion

Our study provided insight in the role of HC, ABA, and ABA-HC for regulating physiological, biochemical and molecular responses during bud quiescence of grape. Exogenous application of HC is an efficient approach to get better understanding of bud quiescence break. Application of HC, ABA, and ABA-HC resulted in a notable increase in APX, POD, and SOD activities, soluble sugars contents and related gene expression. Complete illumination of the roles of HC, ABA during bud quiescence mechanism will be helpful for grape production. Further studies related to our findings may help the underlying mechanism and potential role of HC and ABA during bud quiescence of grape.

## Author Contributions

Conceived and design the experiment: J-MT and MK-U-R Performed the experiments: MK-U-R and WW; Writing of the manuscript: MK-U-R. Analyzed the data: MK-U-R, C-XL, MH, and Y-SX. All authors read and approved the final version of the manuscript.

## Conflict of Interest Statement

The authors declare that the research was conducted in the absence of any commercial or financial relationships that could be construed as a potential conflict of interest.
