# Supplementary figures and images for: Comparative Study on Reagents Involved in Grape Bud Break and Their Effects on Different Metabolites and Related Gene Expression during Winter
Source: Front Plant Sci. 2017 Aug 4;8:1340. doi: 10.3389/fpls.2017.01340 (PMC5543042; doi:10.3389/fpls.2017.01340)

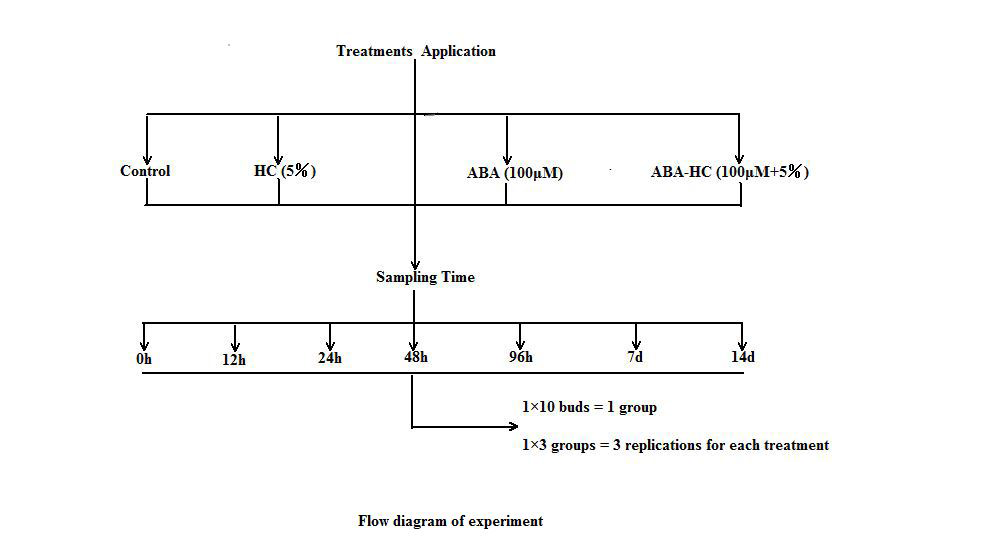

Supplement: Supplementary file 2 [file Image_1.tif]
